# Low Serum 25-hydroxyvitamin D (Vitamin D) Level Is Associated With Susceptibility to COVID-19, Severity, and Mortality: A Systematic Review and Meta-Analysis

**DOI:** 10.3389/fnut.2021.660420

**Published:** 2021-03-29

**Authors:** Mohammad Rizki Akbar, Arief Wibowo, Raymond Pranata, Budi Setiabudiawan

**Affiliations:** ^1^Department of Cardiology and Vascular Medicine, Faculty of Medicine, Universitas Padjadjaran/Dr. Hasan Sadikin General Hospital, Bandung, Indonesia; ^2^Faculty of Medicine, Universitas Pelita Harapan, Tangerang, Indonesia; ^3^Department of Child Health, Faculty of Medicine, Universitas Padjadjaran/Dr. Hasan Sadikin General Hospital, Bandung, Indonesia

**Keywords:** coronavirus, COVID-19, immunity, infection, mortality, severity, susceptibility, vitamin D

## Abstract

**Background:** This systematic review and meta-analysis aimed to assess whether low serum 25-hydroxyvitamin D (25-OHD) level is associated with susceptibility to COVID-19, severity, and mortality related to COVID-19.

**Methods:** Systematic literature searches of PubMed, Scopus, and Embase database up until 9 December 2020. We include published observational prospective and retrospective studies with information on 25-OHD that reported main/secondary outcome. Low serum 25-OHD refers to participants with serum 25-OHD level below a cut-off point ranging from 20 to 30 ng/mL. Other cut-off values were excluded to reduce heterogeneity. The main outcome was mortality defined as non-survivor/death. The secondary outcome was susceptibility and severe COVID-19.

**Results:** There were 14 studies comprising of 999,179 participants. Low serum 25-OHD was associated with higher rate of COVID-19 infection compared to the control group (OR = 2.71 [1.72, 4.29], *p* < 0.001; *I*^2^: 92.6%). Higher rate of severe COVID-19 was observed in patients with low serum 25-OHD (OR = 1.90 [1.24, 2.93], *p* = 0.003; *I*^2^: 55.3%), with a sensitivity of 83%, specificity of 39%, PLR of 1.4, NLR of 0.43, and DOR of 3. Low serum 25-OHD was associated with higher mortality (OR = 3.08 [1.35, 7.00], *p* = 0.011; *I*^2^: 80.3%), with a sensitivity of 85%, specificity of 35%, PLR of 1.3, NLR of 0.44, and DOR of 3. Meta-regression analysis showed that the association between low serum 25-OHD and mortality was affected by male gender (OR = 1.22 [1.08, 1.39], *p* = 0.002), diabetes (OR = 0.88 [0.79, 0.98], *p* = 0.019).

**Conclusion:** Low serum 25-OHD level was associated with COVID-19 infection, severe presentation, and mortality.

## Introduction

Coronavirus disease 2019 (COVID-19) is one of the most prevalent diseases to date (1). Although most COVID-19 patients have mild-moderate symptom, a considerable number of patients, especially in patients with pre-existing comorbidities, experiences severe infection that might lead to death (2, 3).

Vitamin D is known to modulate immune response (4) and its deficiency was associated with respiratory distress in patients hospitalized for pneumonia (5). Nevertheless, controversies exist, a study indicates that low serum 25-hydroxyvitamin D (25-OHD) was not associated with lung injury or mortality in severe sepsis and trauma (6). Numerous studies on vitamin D in COVID-19 patients also have conflicting results, similar to other diseases. This systematic review and meta-analysis aimed to assess whether low serum 25-OHD is associated with susceptibility to COVID-19, severity, and mortality related to COVID-19.

## Materials and Methods

This study follows the Preferred Reporting Items for Systematic Reviews and Meta-Analyses (PRISMA) reporting guideline.

### Eligibility Criteria

The inclusion criteria were: (1) published observational retrospective and prospective studies, (2) Information on serum 25-OHD with a clear cut-off value ranging from 20 to 30 ng/mL, (3) comparing patients with COVID-19 vs non-COVID-19 OR severity in COVID-19 patients OR mortality in COVID-19 patients.

The paper was excluded if it fulfils one of the following: (1) reviews, (2) preprints, (3) non-research letters, (4) case reports, (5) commentaries, and (6) language other than English. We excluded preprints because of inconsistent credibility (7).

### Search Strategy and Study Selection

We performed systematic literature search using PubMed, Scopus, and Embase databases were performed with keywords “COVID-19” OR “SARS-CoV-2” OR “2019-nCoV” AND “Vitamin D” on 9 December 2020.

Duplicate records were removed and the title/abstract was screened by two independent authors. The full-texts of potentially eligible studies were assessed based on the inclusion and exclusion criteria.

### Data Extraction

Two authors independently performed data extraction of first author, publication year, design, age, male (gender), hypertension, diabetes, serum 25-OHD status, the outcome of interest and its effect estimates.

Low serum 25-OHD refers to participants with serum 25-OHD below a cut-off point ranging from 20 to 30 ng/mL. Other cut-off values were excluded to reduce heterogeneity.

The main outcome was mortality defined as non-survivor/death. The secondary outcome was susceptibility and severe COVID-19. Susceptibility was calculated by comparing the COVID-19 positive cohort with the COVID-19 negative cohort. Severe COVID-19 was defined according to the criteria for severe CAP, including the need for intensive unit care or mechanical ventilation (8). The effect estimates of the main and secondary outcome were reported as odds ratios (ORs). Several parameters, including the sensitivity and specificity of low serum 25-OHD, the positive likelihood ratio (PLR) and negative likelihood ratio (NLR), and diagnostic odds ratio (DOR) of studies; and generate hierarchical summary receiver operating characteristic (HSROC) for mortality and severity.

Risk of bias assessment was performed by two independent authors using the Newcastle-Ottawa Scale (NOS). Arising discrepancies were resolved by discussion.

### Statistical Analysis

DerSimonian and Laird random-effects model were used to generate pooled ORs and its 95% CI. *P* ≤ 0.05 was considered as statistically significant. Assessment of heterogeneity was performed by using *I*-squared (*I*^2^) and Cochrane *Q* test, in which an *I*^2^ > 50% or *p* < 0.10 indicates significant heterogeneity. We performed the qualitative funnel plot analysis and the quantitative Egger's test in order to assess the possibility for publication bias and small-study effects. Sensitivity and specificity, PLR and NLR, and DOR were pooled; HSROC was generated. Restricted-maximum likelihood (REML) random-effects meta-regression was performed using age, male (gender), diabetes, and hypertension as covariates. To perform these analyses, STATA 16 (StataCorp LLC, Texas, US) was used.

## Results

**Figure 1:**
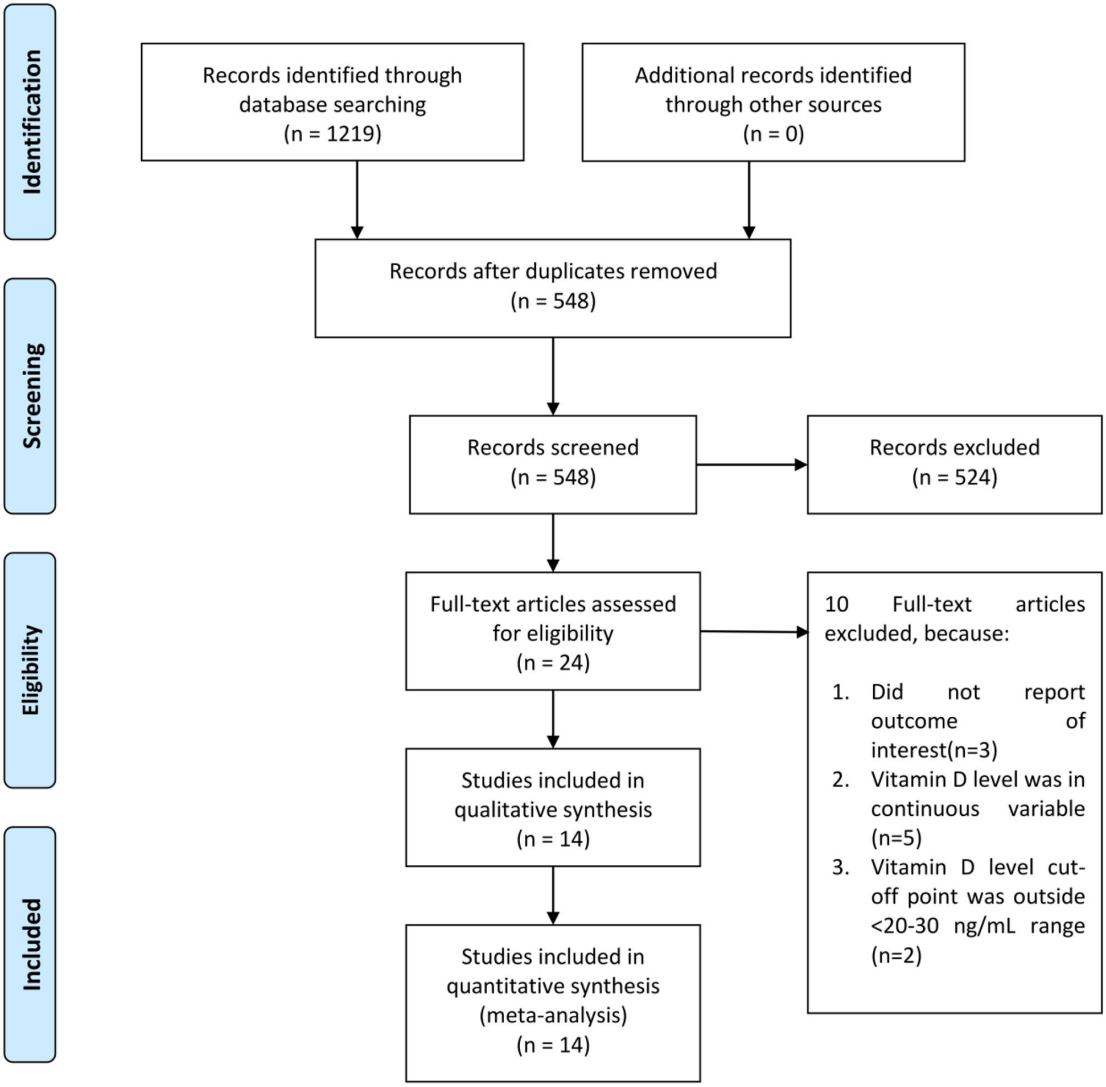
PRISMA flowchart.

There were 14 studies comprising of 999,179 participants in the qualitative and quantitative synthesis (9–22) (Figure 1). The baseline characteristics and risk of bias assessment based on NOS is displayed in Table 1. Severity occurs in 42% (22–62%). Mortality occurs in 24% (6–41%) of patients in the pooled analysis.

**Table 1:** Baseline characteristics of the included studies.

**Authors**	**Design**	**Sample size**	**Vitamin D cut-off (ng/mL)**	**Outcome of interest**	**Age (years)**	**Male (%)**	**Diabetes (%)**	**Hypertension (%)**	**NOS**
Abrishami et al. (10)	Retrospective observational	73	<25	Mortality	55.2	64.4	15.1	24.7	8
Baktash et al. (9)	Retrospective Observational	105	≤ 30	Mortality IMV	81	54.3	32.3	51.4	6
Cereda et al. (22)	Prospective Observational	129	<20	Mortality Severity	77	54.3	30.7	70.1	8
Hastie et al. (21)	Observational	656	<25	Mortality	–	–	–	–	7
Hernández et al. (20)	Restrospective Observational (case-control)	216	<20	Mortality Severity Susceptibility	61	62.4	16.5	40	6
Im et al. (18)	Retrospective Observational	200	<20	Susceptibility	57.5 (COVID-19)	–	–	–	5
Jain et al. (12)	Prospective Observational	154	<20	Mortality	46.1	61.7	–	–	5
Katz et al. (11)	Cross-Sectional	987,849	Deficiency	Susceptibility	Stratified	48.4	–	–	6
		971 (COVID-19)							
Luo et al. (14)	Cross-Sectional	895	<30	Mortality	55.5	45.3	–	–	6
				Severity					
				Susceptibility					
Maghbooli et al. (19)	Retrospective Observational	235	<30	Severity	58.7	61.3	36.6	44.4	6
Meltzer et al. (17)	Retrospective Observational	489	<20	Susceptibility	49.2	25	28	53	8
Merzon et al. (16)	Retrospective Observational	7,807	<20	Susceptibility	44	41.4	–	–	7
		782 (COVID-19)							
adujkovic et al. (15)	Retrospective Observational	185	<20	Mortality	60	51	10	–	8
				Severity					
De Smet et al. (13)	Retrospective Observational	186	<20	Mortality	69	58.6	14	–	8

*IMV, Invasive Mechanical Ventilation; NOS, Newcastle-Ottawa Scale*.

**Figure 2:**
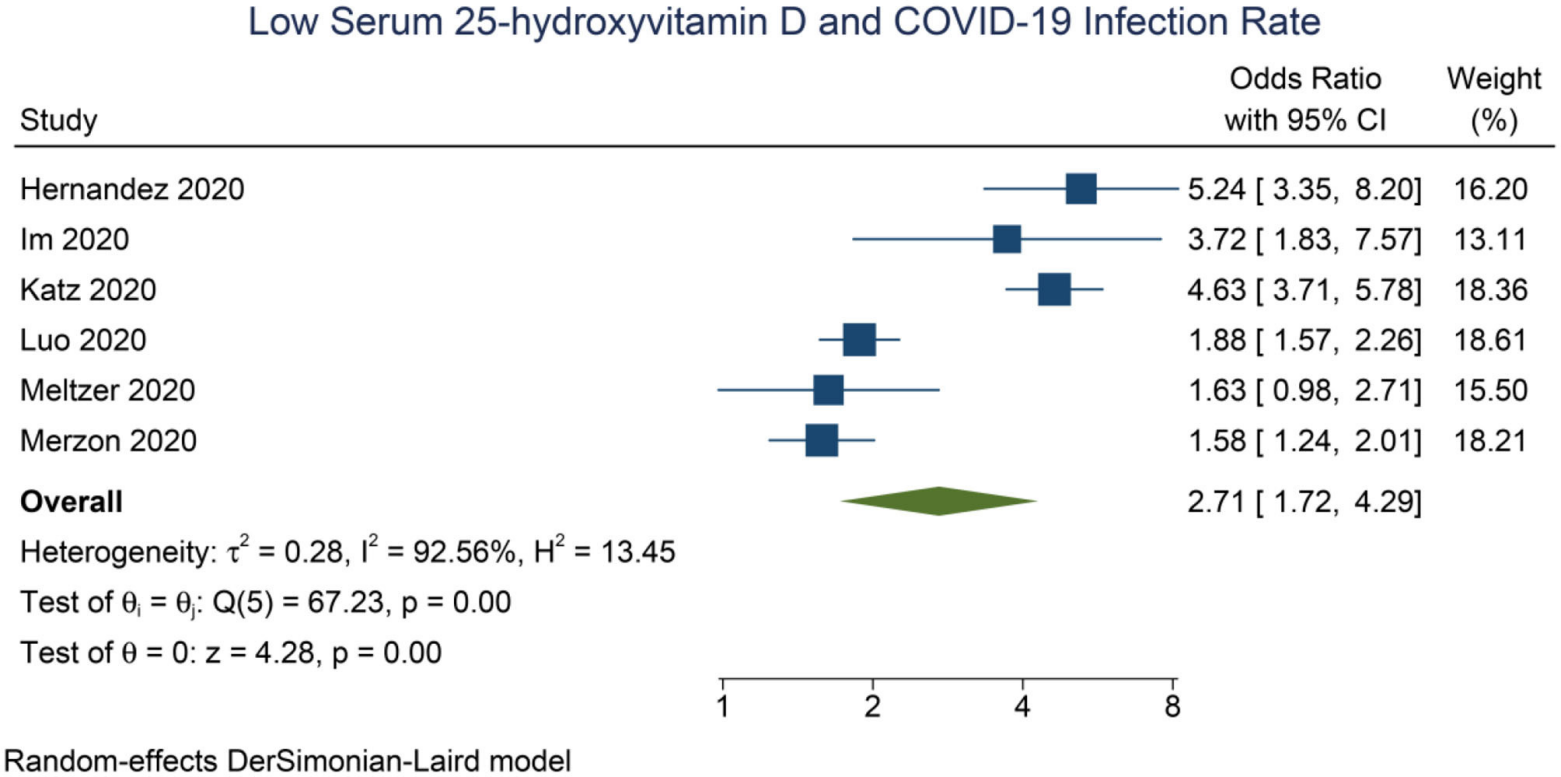
Low Serum 25-hydroxyvitamin D and COVID-19 infection rate.

Meta-analysis showed that low serum 25-OHD was associated with higher rate of COVID-19 infection compared to the control group (OR = 2.71 [1.72, 4.29], *p* < 0.001; *I*^2^: 92.6%, *p* < 0.001) (Figure 2).

**Figure 3:**
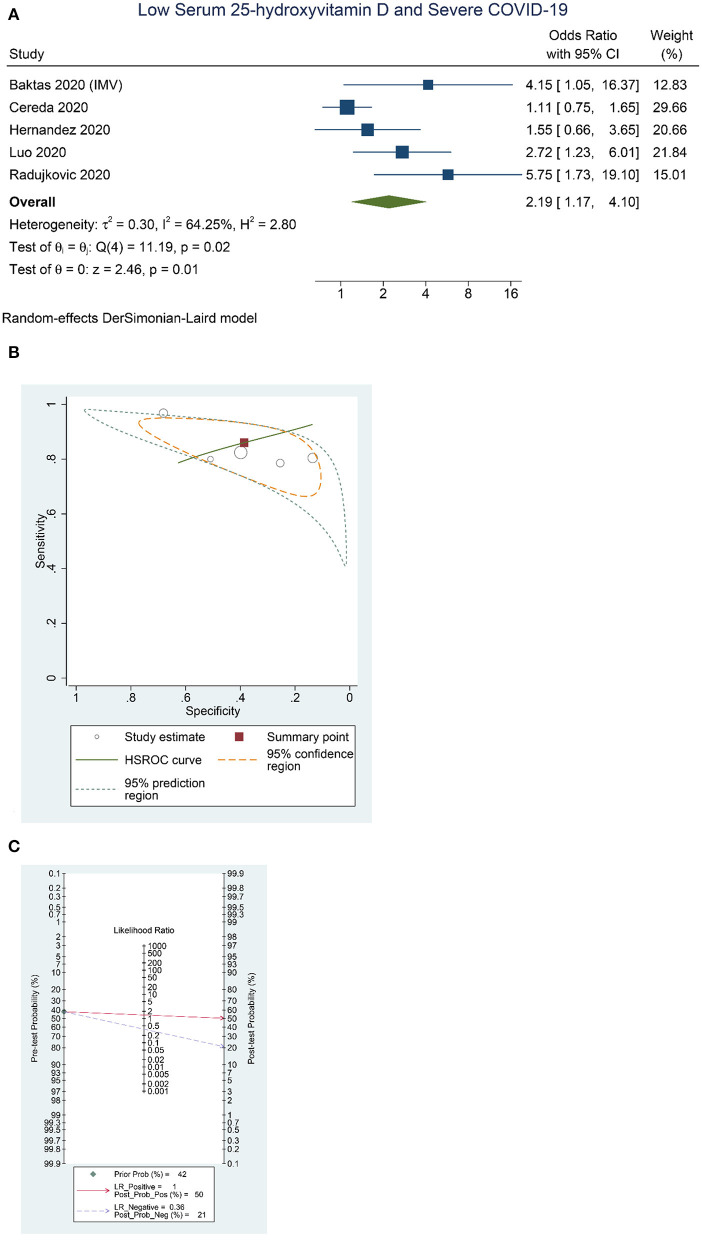
Low Serum 25-hydroxyvitamin D and Severe COVID-19. Forest-plot **(A)**, HSROC curve **(B)**, and Fagan's Nomogram **(C)**. HSROC, hierarchical summary receiver operating characteristic.

Higher rate of severe COVID-19 was observed in patients with low serum 25-OHD (OR = 2.19 [1.17, 4.10], *p* = 0.013; *I*^2^: 64.3%, *p* = 0.025) (Figure 3A), with a sensitivity of 0.86 [0.79, 0.91], specificity of 0.39 [0.23, 0.57], PLR of 1.4 [1.0, 2.0], NLR of 0.36 [0.16, 0.83], and DOR of 4 [1, 12] (Figure 3B). Fagan's nomogram indicate that a low serum 25-OHD was associated with 50% post-test probability and normal serum 25-OHD was associated with 21% post-test probability for mortality, in a sample with 42% pre-test probability (Figure 3C).

**Figure 4:**
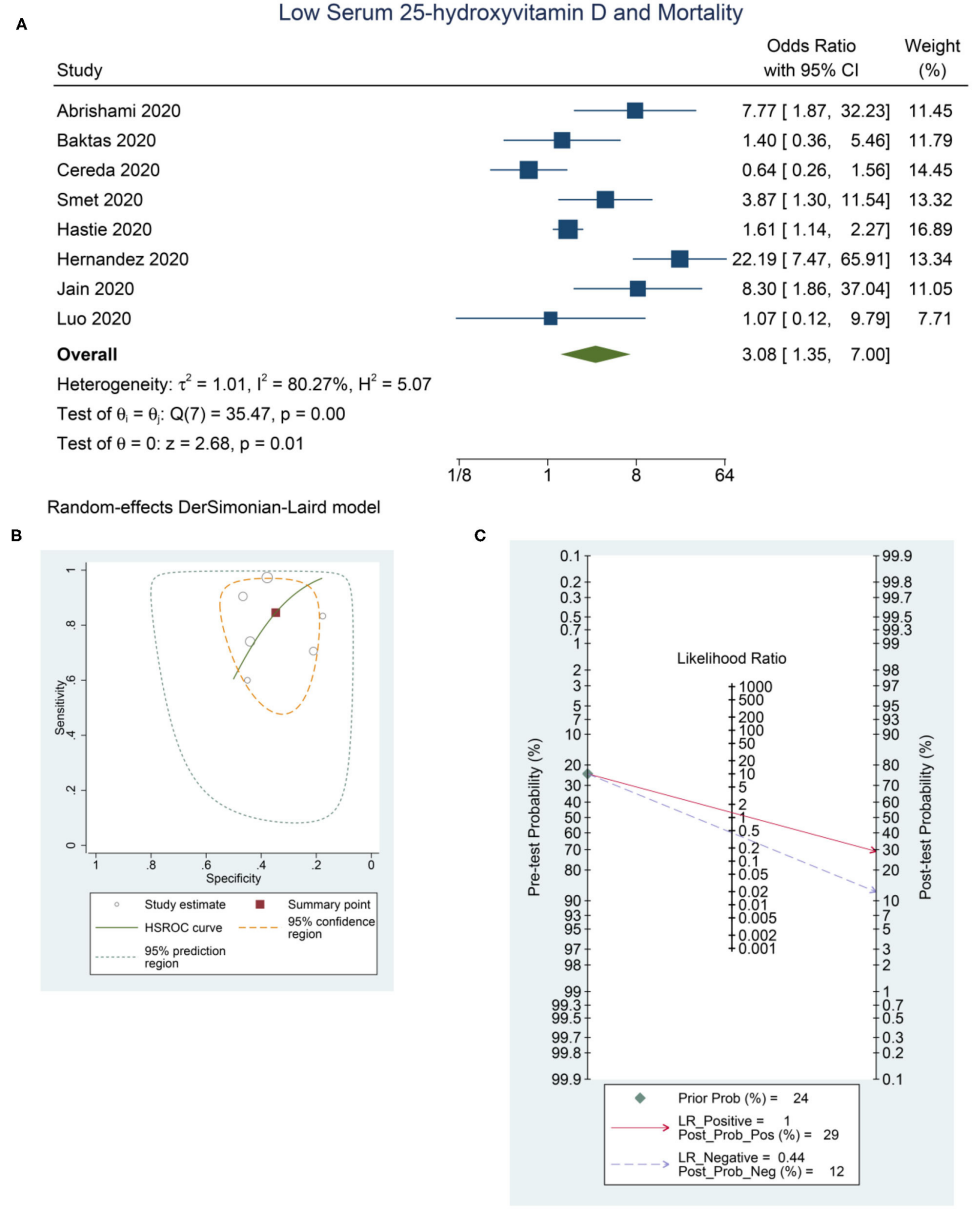
Low Serum 25-hydroxyvitamin D and Mortality. Forest-plot **(A)**, HSROC curve **(B)**, and Fagan's Nomogram **(C)**. HSROC, hierarchical summary receiver operating characteristic.

Low serum 25-OHD was associated with higher mortality (OR = 3.08 [1.35, 7.00], *p* = 0.011; *I*^2^: 80.3%, *p* < 0.001) (Figure 4A), with a sensitivity of 0.85 [0.68, 0.93], specificity of 0.35 [0.26, 0.45], PLR of 1.3 [1.0, 1.6], NLR of 0.44 [0.18, 1.08], and DOR of 3 [1, 9] (Figure 4B). Fagan's nomogram indicate that a low serum 25-OHD was associated with 29% post-test probability and normal serum 25-OHD was associated with 12% post-test probability for mortality, in a sample with 24% pre-test probability (Figure 4C).

Funnel plot was asymmetrical for mortality, severity, and susceptibility. Egger's test indicates significant small-study effects for severity (*p* = 0.047) and mortality (*p* = 0.046). There was no indication of small-study effects for susceptibility (*p* = 0.615).

Meta-regression analysis showed that the association between low serum 25-OHD and COVID-19 infection was affected by age (OR = 1.06 [1.01, 1.12], *p* = 0.020) and male gender (OR = 1.04 [1.00, 1.72], *p* = 0.039).

**Figure 5:**
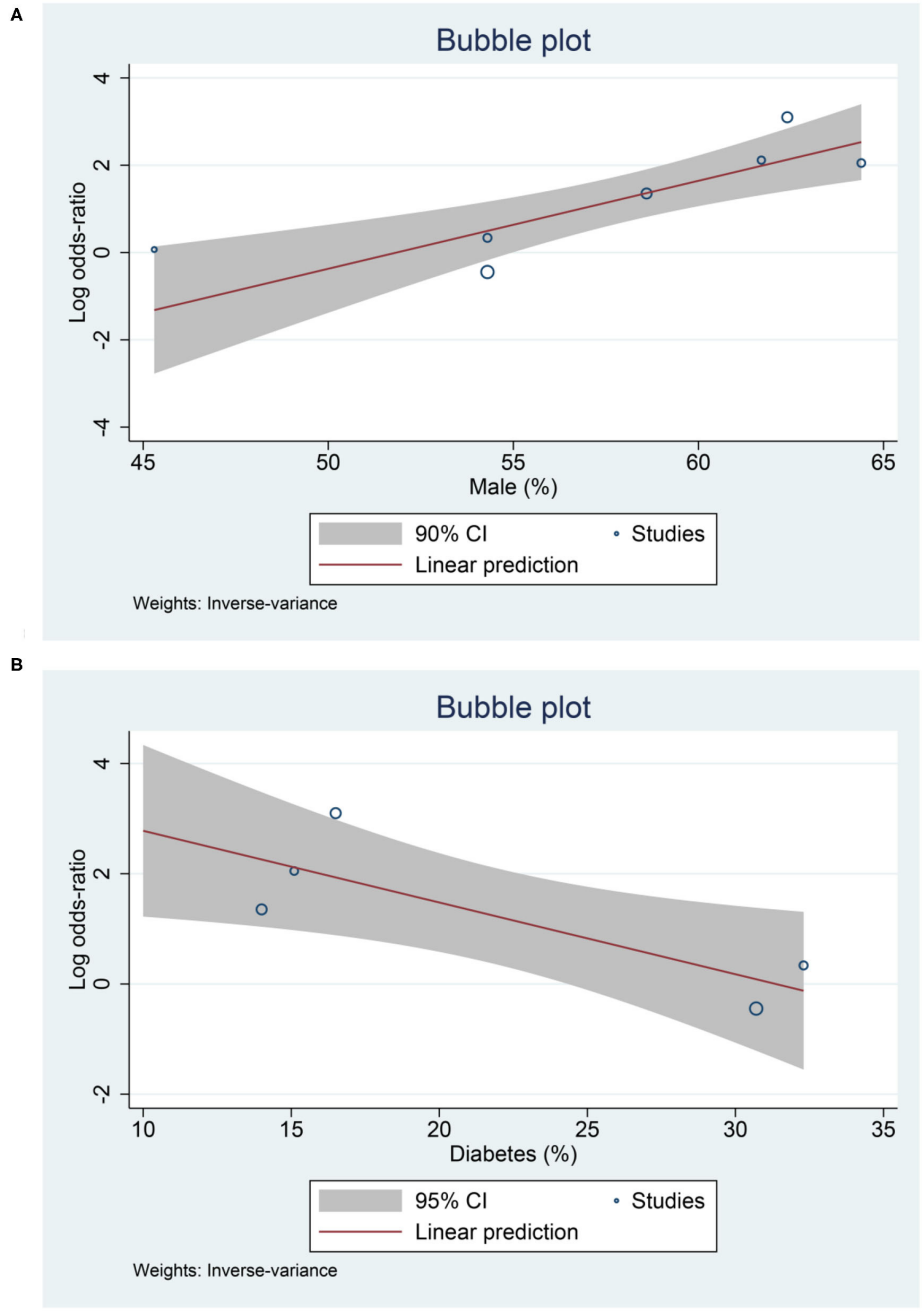
Meta-regression Analysis. Male **(A)** and diabetes **(B)**.

Meta-regression analysis showed that the association between low serum 25-OHD and mortality was affected by male gender (OR = 1.22 [1.08, 1.39], *p* = 0.002) (Figure 5A), diabetes (OR = 0.88 [0.79, 0.98], *p* = 0.019) (Figure 5B); borderline significant for age (OR = 0.93 [0.87, 1.00], *p* = 0.061) and hypertension (OR = 0.93 [0.87, 1.00], *p* = 0.052).

## Discussion

This meta-analysis indicates that low serum 25-OHD levels was associated with higher infection, severe COVID-19, and mortality rate.

Pooled analysis showed that the susceptibility to COVID-19 was higher in patients with low serum 25-OHD. Meta-regression analysis showed that age and gender (male) significantly increase the association. Thus, elderly patients with low serum 25-OHD are more susceptible to COVID-19 compared to non-elderly patients with low serum 25-OHD. Older adults may experience immunosenescence and inflammaging which may affect immune responses against infection, thus contributing to susceptibility (23, 24). Elderly population produces 75% less cutaneous vitamin D3 than young adults and is thus more prone to lower serum 25-OHD level (25). These, in combination, may explain the increased susceptibility to COVID-19 in older adults. Meta-regression showed that male gender affects the association between low serum 25-OHD and susceptibility to COVID-19 and mortality. Vitamin D affects androgen synthesis in testicular cells, and endogenous testosterone may account for differences in properties of low 25-OHD in males and females (26, 27). This may be of special importance in elderly patients. The exact mechanism on why gender plays a role requires further investigation.

The current analysis showed that low serum 25-OHD was associated with higher mortality and severe COVID-19. However, it should be noted that some of studies were excluded because they only provide a comparison of serum 25-OHD levels in a continuous variable, several of these studies showed a no significant difference between mean serum 25-OHD and mortality and severity of COVID-19. A prospective multicenter observational CovILD study of 109 patients by Pizzini et al. (28) indicates that low serum 25-OHD levels at the onset or 8-week follow-up were not associated with persistent symptom burden, lung function impairment, ongoing inflammation, or more severe CT abnormalities. Our study also indicates that the susceptibility to COVID-19 might be higher in patients with low serum 25-OHD, however, a data of 1,326 patients from the UK biobank cohort indicates that Vitamin D level, adjusted with gender, age, and ethnicity was not significant for susceptibility to COVID-19.

Studies evaluating the serum 25-OHD level often have different cut-off points, we included those with 20–30 ng/mL cut-off points. There was a study showed that 10 ng/mL cut-off point was associated with tenfold risk of mortality (29).

Another possible explanation for the association between low serum 25-OHD and poor outcome is that patients with severe illness are often bedridden and have low intake, leading to low serum 25-OHD level (30). Thus subsequent studies need to be able to demonstrate a causal relationship in order to solidify the evidence. Patients with older age and comorbidities such as obesity, diabetes, cardiovascular disease, hypertension, heart failure are also associated with higher mortality in patients with COVID-19 (31–38). Obesity itself was shown to be associated with low serum 25-OHD level (32). The heterogeneity of the pooled effect estimate was high, meta-regression analysis indicates that gender and comorbidities affect the association between low serum 25-OHD and mortality; thus, one of the potential causes of heterogeneity is the variation in the proportion of comorbidities. Thus a well-designed prospective large cohort studies with rigorous statistical analysis and adequate adjustment to covariates are required to obtain the “true” effect of low serum 25-OHD on mortality in COVID-19.

Previously, a meta-analysis showed that Vitamin D supplementation was associated with reduced risk for acute respiratory tract infection (6). Subgroup analysis in the study indicate that vitamin D3 benefit was observed in <800 international units (IU), a statistically non-significant trend in 800–2,000 IU, and no benefit in ≥2,000 IU supplementation. The potential risk of bias should be noted, the observation was made based on five, nine, and eleven studies respectively; with the statistical significance noted in pooling of five studies. Rastogi et al. (39) conducted a randomized controlled study with short term administration of high-dose vitamin D in asymptomatic or mildly symptomatic COVID-19 patients with vitamin D deficiency and noted that a greater proportion of patients turned negative in the vitamin D supplementation group. Additionally, there was a significant decrease in fibrinogen in patients receiving vitamin D. Nonetheless, the study has a small sample size and lacks adjustment for potential confounders on their analysis. These analyses also did not hard endpoints such as mortality or requirement for a more intensive care. Cohort studies have shown potential benefit of vitamin D supplementation in terms of COVID-19 severity (40). A quasi-experimental study indicate possible benefit of vitamin D bolus on survival of COVID-19 patients in a nursing home (41), however, the control arm has only nine patients compared to 57 in the interventional arm. It is unclear why the control group did not receive vitamin D bolus, this might be a potentially important confounder.

### Clinical Implications

Low serum 25-OHD levels was shown to be associated with higher infection rate, severity, and mortality. Whether the relationship is causal remains to be investigated. Vitamin D supplementation is economical and potentially beneficial. Thus, it is recommended to provide supplementation to the Vitamin D deficient patients. Nevertheless, high quality randomized controlled trials are required to determine whether routine Vitamin D supplementation will be useful.

### Limitation

The risk of publication bias, which is caused by positive studies are more likely to be published compared to the negative studies, cannot be ruled out. The retrospective design of the studies presents as a potential source of bias. The cut-off point slightly varies among the pooled analysis. Potential unaccounted confounders may cause bias in the studies, for example, bedridden patients often have low serum 25-OHD compared to the healthy counterpart.

One of the study by Katz et al. (11) has the largest sample size of 987,849. However, we only take Katz et al. study for susceptibility, thus it did not affect the pooled mortality and severity. For the susceptibility analysis, Katz et al. contributes to 18.36% of the weight for susceptibility. Largest contributor is Luo et al. which contributes to 18.61% and the smallest was Im et al. to 13.11%. Thus despite the large sample size compared to the others, it was not expected to change the direction of effect estimate. However, small-study effects in the pooled effect estimate for susceptibility might be caused by the studies with minimal sample because they are weighted almost equally with the larger studies (42). Finally, meta-regression analysis was also based on small number of studies.

In conclusion, low serum 25-OHD level was associated with higher rate of COVID-19 infection, severe presentation, and mortality.

## Data Availability Statement

The original contributions generated for the study are included in the article/supplementary material, further inquiries can be directed to the corresponding author/s.

## Author Contributions

MA: conceptualization, investigation, writing — original draft, writing — review and editing, and supervision. AW: data curation, investigation, and writing — original draft. RP: conceptualization, methodology, software, data curation, formal analysis, statistical analysis, investigation, validation, and writing — original draft. BS: investigation, and writing — review and editing. All authors contributed to the article and approved the submitted version.

## Conflict of Interest

The authors declare that the research was conducted in the absence of any commercial or financial relationships that could be construed as a potential conflict of interest.
