# Corrigendum: Low Serum 25-hydroxyvitamin D (Vitamin D) Level Is Associated With Susceptibility to COVID-19, Severity, and Mortality: A Systematic Review and Meta-Analysis

**DOI:** 10.3389/fnut.2021.754539

**Published:** 2021-09-27

**Authors:** Mohammad Rizki Akbar, Arief Wibowo, Raymond Pranata, Budi Setiabudiawan

**Affiliations:** ^1^Department of Cardiology and Vascular Medicine, Faculty of Medicine, Universitas Padjadjaran/Dr. Hasan Sadikin General Hospital, Bandung, Indonesia; ^2^Faculty of Medicine, Universitas Pelita Harapan, Tangerang, Indonesia; ^3^Department of Child Health, Faculty of Medicine, Universitas Padjadjaran/Dr. Hasan Sadikin General Hospital, Bandung, Indonesia

**Keywords:** coronavirus, COVID-19, immunity, infection, mortality, severity, susceptibility, vitamin D

One of the articles included in our meta-analysis, namely “Vitamin D sufficiency, a serum 25-hydroxyvitamin D at least 30 ng/mL reduced risk for adverse clinical outcomes in patients with COVID-19 infection” by Maghbooli et al. (DATE) has had an Expression of Concern (EoC) published (link to EoC). Consequently, we have redone the analysis and the result confirm our hypothesis. The odds ratio (OR), sensitivity, specificity, positive likelihood ratio (PLR), and diagnostic odds ratio (DOR) actually increases with removal of Maghbooli et al. study (1). Thus, the hypothesis has been confirmed through reanalysis. Below are some changes, with the Maghbooli et al. study excluded (1).

In the original article, there was an error. A correction has been made to **Results**, paragraph 1. The percentage of severity occurring in 47% (27–67%) of patients is incorrect. The corrected paragraph appears below:

“There were 14 studies comprising of 999,179 participants in the qualitative and quantitative synthesis (9–22) (**Figure 1**). The baseline characteristics and risk of bias assessment based on NOS is displayed in **Table 1**. Severity occurs in 42% (22–62%). Mortality occurs in 24% (6–41%) of patients in the pooled analysis.”

In the original article, there were several errors. A correction has been made to **Results**, paragraph 3. The corrected paragraph appears below:

“Higher rate of severe COVID-19 was observed in patients with low serum 25-OHD (OR = 2.19 [1.17, 4.10], *p* = 0.013; *I*^2^: 64.3%, *p* = 0.025) (Figure 3A), with a sensitivity of 0.86 [0.79, 0.91], specificity of 0.39 [0.23, 0.57], PLR of 1.4 [1.0, 2.0], NLR of 0.36 [0.16, 0.83], and DOR of 4 [1, 12] (Figure 3B). Fagan's nomogram indicate that a low serum 25-OHD was associated with 50% post-test probability and normal serum 25-OHD was associated with 21% post-test probability for mortality, in a sample with 42% pre-test probability (Figure 3C).”

**Figure 3:**
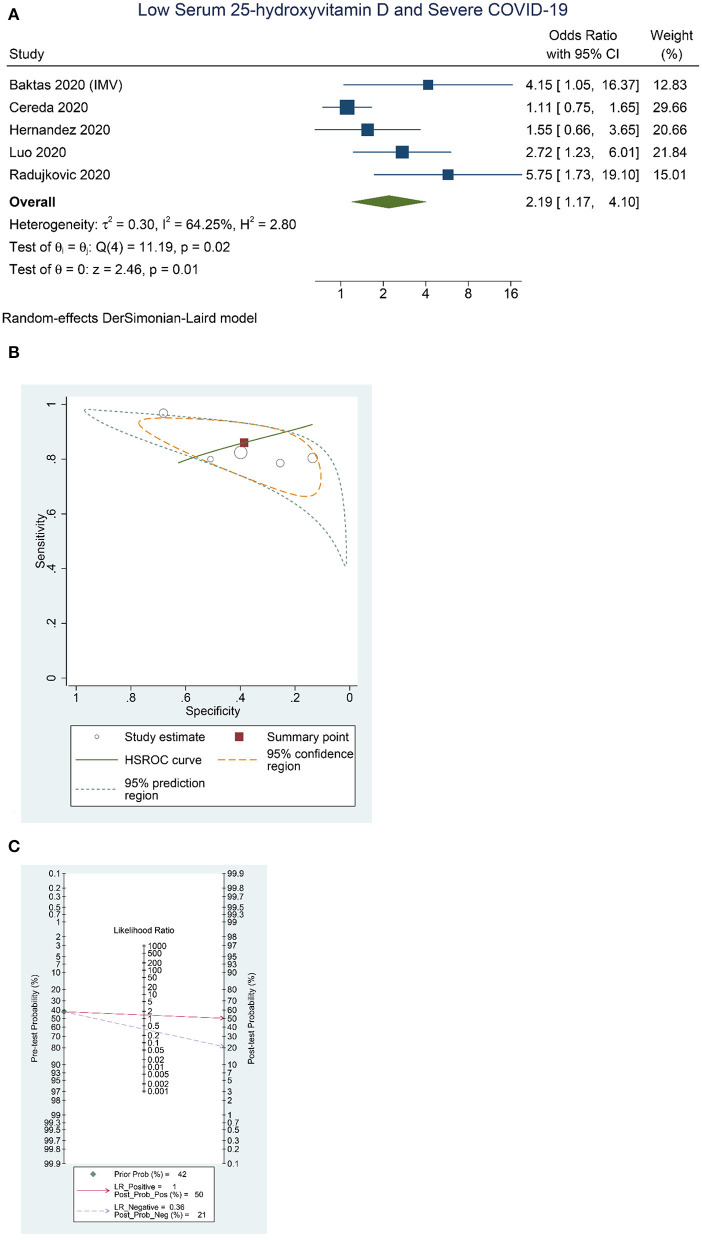
Low Serum 25-hydroxyvitamin D and Severe COVID-19. Forest-plot **(A)**, HSROC curve **(B)**, and Fagan's Nomogram **(C)**. HSROC, hierarchical summary receiver operating characteristic.

Should Maghbooli et al. study be excluded, Figure 3A, Figure 3B, and Figure 3C require updating. The corrected Figure 3A, Figure 3B, and Figure 3C appear below.

The authors apologize for these errors and state that this does not change the scientific conclusions of the article in any way. The original article has been updated.

## Publisher's Note

All claims expressed in this article are solely those of the authors and do not necessarily represent those of their affiliated organizations, or those of the publisher, the editors and the reviewers. Any product that may be evaluated in this article, or claim that may be made by its manufacturer, is not guaranteed or endorsed by the publisher.
